# Diagnostic outcomes of exome sequencing in patients with syndromic or non-syndromic hearing loss

**DOI:** 10.1371/journal.pone.0188578

**Published:** 2018-01-02

**Authors:** Tina Likar, Mensuda Hasanhodžić, Nataša Teran, Aleš Maver, Borut Peterlin, Karin Writzl

**Affiliations:** 1 Clinical Institute of Medical Genetics, University Medical Centre Ljubljana, Ljubljana, Slovenia; 2 Policlinic of Medical Genetics with Genetic Counseling for Out-Patient Care, Department of Paediatrics, University Clinical Centre Tuzla, Tuzla, Bosnia and Herzegovina; Medizinische Universitat Innsbruck Department fur Kinder- und Jugendheilkunde, AUSTRIA

## Abstract

Hereditary hearing loss (HL) is a common sensory disorder, with an incidence of 1–2 per 1000 newborns, and has a genetic etiology in over 50% of cases. It occurs either as part of a syndrome or in isolation and is genetically very heterogeneous which poses a challenge for clinical and molecular diagnosis. We used exome sequencing to seek a genetic cause in a group of 56 subjects (49 probands) with HL: 32 with non-syndromic non-*GJB2* HL and 17 with syndromic HL. Following clinical examination and clinical exome sequencing, an etiological diagnosis was established in 15 probands (15/49; 30%); eight (8/17;47%) from the syndromic group and seven (7/32; 21%) from the non-syndromic non-*GJB2* subgroup. Fourteen different (half of them novel) non-*GJB2* variants causing HL were found in 10 genes (*CHD7*, *HDAC8*, *MITF*, *NEFL*, *OTOF*, *SF3B4*, *SLC26A4*, *TECTA*, *TMPRSS3*, *USH2A*) among 13 probands, confirming the genetic heterogeneity of hereditary HL. Different genetic causes for HL were found in a single family while three probands with apparent syndromic HL were found to have HL as a separate clinical feature, distinct from the complex phenotype. Clinical exome sequencing proved to be an effective tool used to comprehensively address the genetic heterogeneity of HL, to detect clinically unrecognized HL syndromes, and to decipher complex phenotypes in which HL is a separate feature and not part of a syndrome.

## Introduction

Hereditary hearing loss (HL) is one of the most common sensory disorders worldwide, with an incidence of 1–2 per 1000 newborns [1]. In 2004 the World Health Organization reported that over 5% of the world population (cca. 360 million people) had a disabling hearing impediment greater than 40 decibels (measured average for 0.5, 1, 2, 4 kHz (dB)) [2]. Genetic factors account for more than 50% of cases, where the majority exhibit autosomal recessive (AR) inheritance (75–80%) followed by 20–25% autosomal dominant (AD) and 1–1.5% X-linked or of mitochondrial inheritance [3]. It is estimated that just over 200 genes are involved in the process of hearing, which comprises about 1% of all coding genes in the human genome [4].

About 70% of all congenital hereditary HL is non-syndromic (nsHL) [5]. Thus far, 59 autosomal dominant, 78 autosomal recessive and 6 X-linked loci have been identified with 36, 66, and 5 causative genes, respectively [4]. Furthermore, some of these HL genes have been associated with both recessive and dominant forms of HL. There are examples of digenic interactions that cause deafness [1], mitochondrial pathogenic variants as well as certain genetic-environmental interactions that cause HL.

The remaining 30% of HL cases are considered to be syndromic (sHL), wherein the patients exhibit clinical features in at least one other organ system. HL is a feature of between 300 and 400 different syndromes, in which hardness of hearing commonly represents a mild and/or inconsistent feature and many of these syndromes are extremely rare. Nonetheless, HL is also a frequent and/or consistent clinical feature in many syndromes such as Usher syndrome, Pendred syndrome, Waardenburg, Branchio-Oto-Renal and Jarvell, Lange-Nielsen syndromes [6].

This extreme genetic heterogeneity of hereditary nsHL and sHL can often present challenges in its clinical and genetic evaluation. Traditionally, genetic diagnosis of nsHL patients has been carried out for the most common genetic cause of recessive nsHL (the c.35delG variant of the *GJB2* gene) using PCR or Sanger sequencing of the entire *GJB2* gene. Pathogenic variants in this gene are responsible for an estimated 50% of all prelingual, nsHL cases and the carrier rate of the c.35delG variant in the Caucasian population is 1 in 33 [7]. However, considering the great overall number of possible genetic causes for HL, these two methods have proven to be inefficient, costly and very time consuming when dealing with cascade sequencing of coding regions in a large number of genes. Recently, with the advent of next generation sequencing (NGS) in medical molecular laboratories, high-throughput sequencing of a large set of HL-associated genes has become possible. Massively parallel sequencing of a large number of exons in a single experiment has increased the diagnostic yield and contributed to better characterization of known genes associated with HL [8,9].

The majority of studies published (reviewed in [10]) on the usage of NGS technologies for genetic testing of HL included only individuals with nsHL and have used panels with different types and numbers of genes, while data on the utility of exome sequencing and the utility of NGS technologies in individuals with sHL are sparse.

In the present work we used exome sequencing to seek a genetic cause in a group of 56 patients with either syndromic HL or non-syndromic non-*GJB2* HL. This work illustrates genetic heterogeneity of HL and added value of exome sequencing approach in patients with complex phenotypes.

## Materials and methods

### Patients

This study was carried out at the Clinical Institute of Medical Genetics at the University Medical Center of Ljubljana, Slovenia and the University Medical Center in Tuzla, Bosnia and Herzegovina (BiH). It was approved by the National Medical Ethics Committee of the Republic of Slovenia (No.98/05/12). All participants provided written informed consent prior to enrolment in our study. Written informed consent for all child participants was obtained on their behalf from their parents.

Fifty-six individuals with HL were recruited for our study, 30 from Slovenia and 26 from BiH, all of Caucasian origin. The inclusion criteria were: bilateral, pre-lingual HL with a negative medical history related to potential causes of acquired HL (infection, trauma, ototoxic drugs, perinatal complications). Brain stem evoked acoustic potentials and pure-tone audiometry were used to assess the degree and progression of HL. Hearing loss severity was classified as mild (26–40 dB), moderate (41–55 dB), moderately severe (56–70 dB), severe (71–90 dB), or profound (>91 dB) [11]. All individuals routinely underwent neurological and ophthalmological examination in the patients’ country of origin (Slovenia or BiH).

Fifty-six individuals belonging to 48 unrelated families were in our HL cohort. Siblings and parents of probands were not considered for diagnostic yield calculation, except in one case where two members of the same family had different genetic causes of HL. Additional family members were recruited for co-segregation analysis whenever possible.

Fourty-nine probands were divided into two subgroups based on their clinical presentation; the nsHL subgroup of 32 probands with no other clinical findings observed at the time of clinical examination, and the sHL subgroup of 17 probands having at least one additional clinical finding besides HL (S1 Table).

All probands from the non-syndromic subgroup were prescreened by conventional Sanger sequencing for pathogenic variants in *GJB2*, while all probands from syndromic subgroup underwent clinical exome sequencing as the first genetic test.

### Exome sequencing

Clinical exome sequencing (cES) was performed using the in-solution capture of exonic sequences with Nextera Rapid Capture Enrichment kit (Illumina, USA) targeting the exons of 4813 genes associated with human genetic disease (TruSight One Panel by Illumina, USA). Sequencing was performed on the Illumina MiSeq platform in 2 x 100 pair-end reads. Raw sequence files were processed using a custom in-house exome analysis pipeline, based on a GATK best practices backbone. Alignment of reads to the human reference assembly (hg19) was performed using the Burrows-Wheeler (BWA) aligner, duplicate sequences removed using Picard MarkDuplicates, followed by base quality score recalibration, variant calling, variant quality score recalibration and variant filtering using elements of the GATK toolset [12].

### Variant analysis and filtration

Variants were stored and annotated in the variant collection and annotation system, based on vtools and ANNOVAR software. Refseq gene models were used for transcript positioning of variants and annotations from dbSNP v138 were used for single nucleotide polymorphism (SNP) annotation. The Slovene genomic variation database, based on a compilation of 1500 exomes was considered the primary source for assessment of variants’ prevalence in the population. Furthermore, the datasets of the Exome Aggregation Consortium (ExAC, exac.broadinstitute.org), UK10K control population (www.uk10k.org) and GoNL (www.nlgenome.nl) projects were employed as sources of variant frequencies in other worldwide populations. Consensus calls of dbNSFP v2 precomputed pathogenicity predictions were used for evaluation of pathogenicity for missense variants. Additionally, SNPeff predictors were utilized as a means of parallel annotation of variant effects. GERP++ rejected substation (RS) scores were used as the fundamental information source of evolutionary sequence conservation. Our pipeline included ClinVar, HGMD (http://www.hgmd.cf.ac.uk/ac/index.php), LOVD (http://www.lovd.nl/3.0/home) and Hereditary Hearing loss Homepage databases as sources of known disease association for identified variants.

The search for causative variants was first focused on genes already associated with HL (S2 Table). In the case of syndromic HL patients, we surveyed the variants in genes associated with syndromic features that accompanied the hearing impairment. The associations were tracked by the Human Phenotype Ontology database (http://human-phenotype-ontology.github.io/). We supplemented this list of genes with genes in deafness gene panels [13].

A minimum median coverage of 60x was required to proceed with the interpretation of exome sequencing data. Variants were taken into consideration, if they were covered by at least 5 reads and if the GATK variant call quality score exceeded 100.0.

We filtered the variants in accordance with the mode of inheritance, variant functional effect (we considered missense, nonsense, splice site, in-frame INDELs and frame-shift INDELs in our analysis) and by masking the variant set with phenotype gene panels. Considering the relatively high frequency of more prevalent deafness-associated variants in the general population, we used relaxed frequency threshold criteria for variant selection. For variants in genes, associated with dominant inheritance, we filtered out variants attaining a frequency above 0.01% in control. Conversely, for variants in genes, associated with recessive inheritance we excluded the variants with a minor allele frequency above 2% in the general population.

### Variant classification and validation

All variants were classified according to the guidelines from the American College of Medical Genetics to pathogenic, likely pathogenic, of uncertain significance, likely benign or benign [14], and novel variants were submitted to the ClinVar Database (https://www.ncbi.nlm.nih.gov/clinvar/). Pathogenic and likely pathogenic variants were classified as disease causing variants.

Candidate variants found by NGS were validated using Sanger sequencing if the coverage at the variant site of the exome sequencing result was below 30x and/or the base quality score below 500, in accordance with previously published recommendations [15]. Furthermore, Sanger sequencing was employed to resolve cases with a suspected compound heterozygous combination of variants and for other familial segregation analyses. Sequencing was carried out using BigDye 3.1 sequencing chemistry (Life Technologies), followed by capillary electrophoresis on the ABI 3500 capillary sequencer (Life Technologies).

Primer sequences are available upon request.

## Results

### Study group

Of the 49 probands included in our study 32 patients were deemed non-syndromic and 17 syndromic, according to their clinical data and their family’s medical history. The mean age of the probands at the start of the study was 8.25 years in the non-syndromic group (with a median of 7 and an age range from 2 to 36 years) and 9.5 years in the syndromic group (with a median of 8 and an age range of 1 to 35 years). Familial and sporadic cases were included and probands had prelingual, bilateral, mild to profound HL. Details of the study group are presented in Table 1.

**Table 1:** Primary cohort characteristics (n = 49 probands).

	Non-syndromic group		Syndromic group	
Probands	All (N = 32)	Probands with PV or VUS (N = 10)	All (N = 17)	Probands with PV or VUS (N = 9)
Female	17	5	9	5
Male	15	5	8	4
*Family history*				
Familial	10	5	4	2
Sporadic	22	5	13	7
*Level of hearing loss*				
Mild	2	0	2	2
Moderate	2	2	5	2
Moderate-Severe	10	3	5	3
Severe	9	2	1	1
Profound	9	3	4	1

PV: pathogenic or likely pathogenic variant; VUS: variant of uncertain significance.

### Variant spectrum

Twenty rare non-*GJB2* variants were found in 15 genes. We identified a single variant in 13 genes (*CHD7*, *HDAC8*, *MIR96*, *MITF*, *MYH14*, *NEFL*, *RYR1*, *SF3B4 TECTA*, *TMC1*, *TMPRSS3*, *USH2A*, and *WFS1*), two variants in *OTOF* and five variants in *SLC26A4* (Table 2). Fifteen variants were classified as pathogenic or likely pathogenic, considered disease-causing variants, and five as variants of unknown significance (*MIR96*, *MYH14*, *TMC1*, *WFS1*, and one of five *SLC26A4* variants). In total, 53% (8/15) of the disease-causing variants were novel.

**Table 2:** Disease causing variants and variants of uncertain significance in HL probands.

Patient ID	Gene	Nucleotide Change	Zygosity	Inh	Mutation type	Protein Change	Variant effect predictors			ExAC (N)	Novel variant	Citation
							SIFT	PolyPhen2	Mutation Taster			
**Syndromic HL group**												
P794	*CHD7*	c.6892C>T	Het	AD	nonsense	p.Gln2298*	/	/	A	0	Yes	ClinVar: ID 374090
P152	*HDAC8*	c.522C>A	Het	AD	nonsense	p.Tyr174*	/	/	A	0	Yes	ClinVar: ID 446297
P045; P2091	*MITF*	c.943C>T	Het	AD	nonsense	p.Arg315*	/	/	A	0	No	ClinVar: ID 14276; [16]; [17]; [18]
P584	*NEFL*	c.293A>G	Het	AD	missense	p.Asn98Ser	/	/	/	0	No	ClinVar: ID 41236
P144	*RYR1*	c.7111G>A	Het	AD	missense	p.Glu2371Lys	D	PD	DC	0	Yes	ClinVar: ID 374164
P552	*SF3B4*	c.827delC	Het	AD	frameshift	p.Pro276fs	/	/	/	0	Yes	ClinVar: ID 446295
P074	*TECTA*	c.6061C>T	Het	AD	missense	p.Arg2021Cys	D	PD	DC	0	Yes	(c.6062G>A; p.Arg2021His)[19]
P354	*MIR96*	**n.43G>A	Het	AD	non-coding transcript variant	/	/	/	/	2/8758	No	(n.42C>T) [20]
P554	*TMC1*	**c.1141T>A	Het	AD	missense	p.Tyr381Asn	D	PD	DC	5/66712	No	ClinVar: ID 229314 (reported in controls) [21]
P476	*MYH14*	**c.5105T>C	Het	AD	missense	p.Val1702Ala	T	B	/	4/59286	No	ClinVar: ID373967
	*WFS1*	**c.2437G>A	Het	AD	missense	p.Val813Met	D	PD	DC	2/65168	No	ClinVar: ID373968
**Non-Syndromic HL group**												
P314	*GJB2*	c.35delG	C Het	AR	frameshift	p.Gly12Valfs*2	/	/	A	585/66686	No	ClinVar: ID 17004; [22]
		c.269T>C	C Het	AR	missense	p.Leu90Pro	D	PD		101/66708	No	ClinVar: ID 17016; [23]
P555	*GJB2*	c.35delG	Hom	AR	frameshift	p.Gly12Valfs*2	/	/	A	585/66686	No	ClinVar: ID 17004; [22]
P162	*OTOF*	c.2677-2A>G	C Het	AR	splice and intron variant	/	/	/	DC	0	Yes	ClinVar: ID 374018
		c.4483C>T	C Het	AR	nonsense	p.Arg1495*	/	/	A	0	No	ClinVar: ID 65804; [24]
P976	*SLC26A4*	c.299T>C (pat)	C Het	AR	missense	p.Leu100Pro	T	PD	DC	0	Yes	ClinVar: ID373979
		c.1693T>G (mat)	C Het	AR	missense	p.Cys565Gly	T	B	DC	0	Yes	ClinVar: ID373978 c.1694G>A (p.Cys565Tyr; ClinVar: ID 43519)
		**c.1730T>C (mat)		AR	missense	p.Val577Ala	D	PD	DC	2/66624	No	ClinVar: ID 94601
P564	*SLC26A4*	c.1003T>C	C Het	AR	missense	p.Phe335Leu	T	PD	/	54/66716	No	ClinVar: ID 4842; [25]; [26]
		c.1790T>C	C Het	AR	missense	p.Leu597Ser	D	PD	/	498/66564	No	ClinVar: ID 43525; [25]; [26]
P694	*TMPRSS3*	c.208delC	Hom	AR	frameshift	p.His208Thrfs*	/	/	A	49/66068	No	ClinVar: ID 165492
P584; P852	*USH2A*	c.11864G>A	Hom	AR	nonsense	p.Trp3955*	/	/	A	12/66762	No	ClinVar: ID 2357; [27]; [28]

A, disease causing automatic. AD, autosomal dominant. AR, autosomal recessive. B, benign. C Het, compound heterozygosity, D, damaging. DC, disease causing. Het, heterozygous. Hom, homozygous. Inh, inheritance. PD, probably damaging. T, tolerated. ExAC, European (Non-Finnish) Population.

**variant of uncertain significance.

Seven of the 49 probands presented with a heterozygous disease-causing variant resulting in dominant HL (*CHD7*, *HDAC8*, *NEFL*, *SF3B4*, *TECTA*, and twice in *MITF)*. The pedigrees were consistent with dominant HL (S1 Fig). The *de novo* origin of the variant was confirmed in sporadic cases with novel missense variants, and segregation was analyzed in familial cases.

In eight of the 49 probands, we identified a homozygous or compound heterozygous disease-causing variant in a gene associated with recessive HL (*OTOF*, *TMPRSS3*, and twice in *GJB2*, *SLC26A4*, and *USH2A*). The group comprised of five sporadic (*GJB2*, *OTOF*, *USH2A*, and *SLC26A4* twice) and three familial cases (*GJB2*, *TMPRSS3*, *USH2A*) (S1 Fig). Both probands with *GJB2* pathogenic variants were originally assigned to the suspected syndromic HL subgroup. This was due to proband P314 having several additional clinical features (see Table 3) and in the case of proband P555 as a result of her family history of HL and retinitis pigmentosa in addition to her infancy (1 year of age) at the time of examination.

**Table 3:** Clinical characteristics of HL probands with rare variants.

Proband ID	HL	Nucleotide	Zygosity	Inh	Phenotype	Final diagnosis
**Nonsyndromic HL**						
P354	fam	*MIR96*:NR_029614.1:n.43G>A	Het	AD	*Moderately severe SNHL*	Nonsyndromic SNHL
P2091	fam	*MITF*:NM_198159.2:c.943C>T	Het	AD	*Severe-profound SNHL*	Waardenburg syndrome
P074	fam	*TECTA*:NM_005422.2:c.6061C>T	Het	AD	*Moderately severe SNHL; mild ID*	Nonsyndromic SNHL, DFNA12
P554	fam	*TMC1*:NM_138691.2:c.1141T>A	Het	AD	*Mild to moderate SNHL between 0*.*1 and 1kHz*, *then steeply sloping to severe to profound HL between 1 and 4KHz*.	Nonsyndromic SNHL
P476	spor	*MYH14*: NM_001145809.1:c.5105T>C; *WFS1*: NM_001145853.1:c.2437G>A	Het; Het	AD	*Moderately severe SNHL*	Nonsyndromic SNHL
P162	spor	*OTOF*:NM_194248.2:c.4483C>T*; OTOF*:NM_194248.2:c.2677-2A>G	Het; Het	AR	*Profound SNHL*	Nonsyndromic SNHL, DFNB9
P564	spor	*SLC26A4*:NM_000441.1:c.1003T>C*; SLC26A4*:NM_000441.1:c.1790T>C	Het; Het	AR	*Severe SNHL; Mondini malformation*	Nonsyndromic SNHL, DFNB4
P976	*spor*	*SLC26A4*:NM_000441.1:c.299T>C*; SLC26A4*:NM_000441.1:c.1693T>G*; SLC26A4*:NM_000441.1:c.1730T>C	Het; Het; Het	AR	*Sudden deterioration of hearing at the age of 18 months followed by phases of fluctuating HL leading to profound SNHL*	Nonsyndromic SNHL, DFNB4
P694	fam	*TMPRSS3*:NM_024022.2:c.208delC	Hom	AR	*Profound SNHL*	Nonsyndromic SNHL
P584	spor	*USH2A*:NM_206933.2:c.11864G>A	Hom	AR	*Moderate SNHL*	Usher syndrome type 2A
**Syndromic HL**						
P794	spor	*CHD7*:NM_017780.3:c.6892C>T	Het	AD	*Progressive mixed*, *bilateral HL; SNHL component profound; middle and inner ear anomalies; myopia*, *bilateral retinal coloboma*, *hypothyroidism; coeliac disease; above average IQ*	CHARGE syndrome
P152	spor	*HDAC8*:NM_018486.2:c.522C>A	Het	AD	*Moderate SNHL; global developmental delay*, *seizures*, *microcephaly*, *dysplastic facial features*, *fingers and toes syndactyly*	Cornelia de Lange syndrome
P045	spor	*MITF*:NM_198159.2:c.943C>T	Het	AD	*Severe SNHL; unilateral heterochromia*, *dorsiflexion of 2nd toes*	Waardenburg syndrome
P584	spor	*NEFL*:NM_006158.3:c.293A>G	Het	AD	*Mild SNHL between 0*.*125 kHz and 4 KHz*, *moderate HL between 4 KHz and 8 KHz; delayed motoric milestones*, *difficulties in walking*, *progressive distal weakness of the lower and upper limbs*, *cerebellar dysfunction*, *peripheral motor and sensory neuropathy*	Charcot-Marie Tooth Disease 2E/1F
P144	spor	*RYR1*:NM_000540.2:c.7111G>A	Het	AD	*Mild SNHL between 6 kHZ and 8 kHz; congenital arthrogryposis*, *delayed motoric milestones*, *severe scoliosis*	Central Core disease
P552	spor	*SF3B4*:NM_005850.4:c.827delC	Het	AD	*Moderately severe conductive hearing loss; malar hypoplasia*, *micrognathia*, *thumb hypoplasia*, *radioulnar synostosis*	NAGER syndrome
P555	fam	*GJB2*:NM_004004.5:c.35delG	Hom	AR	*Moderately severe SNHL; both parents are hearing impaired*, *father also has RP along with some of his other close relatives*	Nonsyndromic SNHL
P314	spor	*GJB2*:NM_004004.5:c.35delG*; GJB2*:NM_004004.5:c.269T>C	Het; Het	AR	*Moderately severe SNHL; microphthalmia*, *solitary kidney*, *omphalocele*, *dysplastic facial features; normal growth and development*	Nonsyndromic SNHL
P852	fam	*USH2A*:NM_206933.2:c.11864G>A	Hom	AR	*Moderate SNHL; retinitis pigmentosa*	Usher syndrome 2A

AD, autosomal dominant. AR, autosomal recessive. Fam, familial. Het, heterozygous. HL, hearing loss. Hom, homozygous. ID, intellectual disability. Inh, inheritance. SNHL, sensorineural hearing loss. Spor, sporadic.

**Fig 1:**
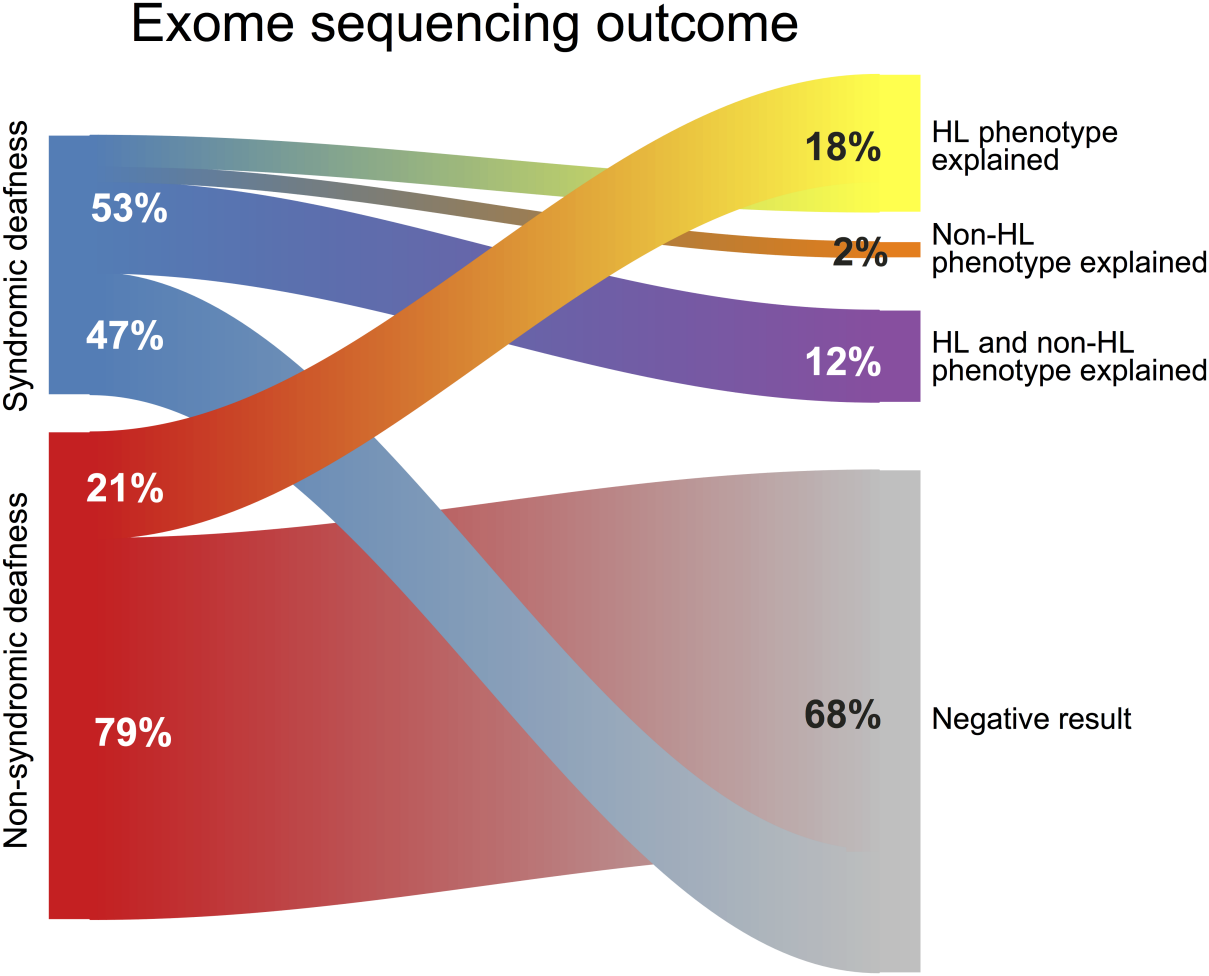
Exome sequencing outcome in the cohort of probands with non-syndromic and syndromic HL.

The overall diagnostic yield of the HL cohort was 30.6% (15/49). The diagnostic yield of the syndromic subgroup (47%; 8/17) was higher than that of the non-syndromic non-*GJB2* HL subgroup (21.8%; 7/32) (Fig 1). Of the 17 individuals in the syndromic subgroup, nine (52.9%) had positive genetic testing results. With the use of cES, we were able to identify whether HL was, indeed, part of a syndromic form of HL or whether it was a separate feature of a complex phenotype. We found genetic causes for syndromic forms of HL in six probands, and genetic variants causing either a non-syndromic HL or a non-HL phenotype in three patients. Of the later, two probands (P555 and P314) had pathogenic variants causing the HL phenotype in *GJB2*, while one proband (P144) was found to have a variant for Central Core Disease, a non-HL phenotype.

### Genotype-phenotype associations

Eight novel likely pathogenic variants were identified in the HL cohort. Seven of them were causal for HL and one for a non-HL phenotype. The characteristics of these genetic variants are presented in Table 2 and genotype-phenotype associations are described in Table 3. S1 Fig. depicts segregation patterns.

Four variants were frameshift, nonsense or splice-site (in *CHD7*, *HDAC8*, *SF3B4*, and *OTOF*) mutations and therefore highly likely to be pathogenic.

#### CHD7

A novel heterozygous nonsense variant: c.6892C>T, p.(Gln2298*). *CHD7* pathogenic variants are known to be associated with CHARGE (Coloboma, Heart, Choanal Atresia, Retardation and Ear Anomalies) syndrome (OMIM 214800). The proband had mixed conductive and sensorineural progressive HL leading to profound HL before the age of 20. He presented with normal growth and had received a master degree; however he had bilateral retinal coloboma and ear anomalies consistent with CHARGE syndrome.

#### HDAC8

A novel heterozygous nonsense variant: c.522C>A, p.(Tyr174*) was found in a 10-year-old proband (P152), who had bilateral, moderate, congenital sensorineural HL (SNHL). She presented with developmental delay, a severe speech impediment, intellectual disability, and seizures. Clinical investigation revealed microcephaly, arched eyebrows, synophrys, pectus excavatum, partial skin syndactyly of fingers 3 and 4 and syndactyly of the second and middle toes. The hand X-rays showed short 5^th^ metacarpal bones bilaterally. The proband’s clinical features were consistent with the clinical diagnosis of Cornelia de Lange syndrome, a clinical and genetically heterogeneous disorder, caused by heterozygous pathogenic variants in *HDAC8* among others [29], [30].

#### SF3B4

A novel *de novo* heterozygous frameshift variant: c.827delC, p.(Pro276fs) was detected in proband P552, who had moderately severe congenital conductive bilateral HL of 50–90 dB with a peak at 1kHz. He presented with facial anomalies (malar hypoplasia and severe microganthia), and limb anomalies (bilateral hypoplasia of the thumbs and unilateral proximal radioulnar synostosis). Heterozygous loss-of-function variants in *SF3B4* are a known cause of Nager syndrome, which is consistent with the patient’s described clinical features.

#### OTOF

Compound heterozygous variants were found in the *OTOF* gene in the proband P162. The variant c.2677-2A>G was novel, whereas the c.4483C>T, p.(Arg1495*) variant was a known pathogenic one (ClinVar 65804). Proband P162 presented with congenital bilateral profound SNHL, which is characteristic for *OTOF*-related non-syndromic HL.

Rare novel disease-causing missense variants were identified in three genes: in *SLC26A4* and *TECTA* explaining the cause of HL and in *RYR1* identifying the non-HL phenotype.

#### SLC26A4

Proband P976 had three rare missense variants; compound heterozygous novel variants c.299T>C, p.(Leu100Pro) and c.1693T>G, p.(Cys565Gly) were predicted to be disease-causing, whereas the c.1730T>C variant has already been reported as a variant of unknown significance (ClinVar 194601). Proband P976 presented with abrupt bilateral SNHL at the age of 18 months, followed by phases of fluctuating HL, finally leading to profound HL. This phenotype is characteristic for HL due to pathogenic variants in *SLC26A4*. Thyroid function was normal.

#### TECTA

The heterozygous novel variant c.6061C>T, p.(Arg2021Cys) is located in the zona pellucida domain of TECTA, where several missense variants have been reported to be associated with dominant forms of HL [31], one of them altering the same amino acid (p.Arg2021His in Iwasaki et al. [19]). Proband P074 and affected sibling shared the same disease-causing variant, which was not present in the normal-hearing mother and was likely inherited from the affected father who was not available for testing. Siblings presented with congenital, non-progressive, bilateral, symmetrical HL of 50-70dB and 60-80dB that is consistent with DFNA8.

#### RYR1

The *de novo* heterozygous novel variant c.7111G>A in exon 44 (p.(Glu2371Lys) was located within the mutational “hotspot” of domain 2 (exons 39–46) [32]. Pathogenic variants in *RYR1* are known to be associated with autosomal dominant and autosomal recessive central core disease (CCD) (OMIM 117000), and malignant hyperthermia (OMIM 145600) but have not been associated with HL. Proband P144 suffered from bilateral, high frequency (6–8 kHz), mild (25dB) SNHL, which is not known to be part of CCD. The proband also presented with congenital arthrogryposis, delayed motor milestones with walking achieved at the age of 3 years, and severe scoliosis first noticed at the age of 4 years and requiring first surgical correction at the age of 12 years. This phenotype was consistent with autosomal dominant CCD, caused by a *RYR1* mutation.

Five variants (in *MIR96*, *TMC1*, *MYH14*, *WFS1*, and *SLC26A4*) were classified as variants of unknown significance (Tables 2 and 3).

## Discussion

We conducted comprehensive gene analysis using clinical exome sequencing in a group of probands with non-syndromic non-*GJB2* HL and in a group with syndromic HL from the Slovene and Bosnian populations. The strength of our approach is the inclusion of individuals with syndromic HL and the usage of a clinical exome instead of targeted panels, which enabled us to identify not only the cause of non-syndromic and syndromic HL phenotypes but also to decipher whether HL is part of a syndrome or a separate clinical feature.

There are three important findings from our study.

First, we found the genetic cause for HL in 15 of the 49 probands evaluated (30%). These included individuals with nsHL and individuals with apparently sHL. In the subgroup of nsHL, the diagnostic yield reached 21.8% (7/32). It should be noted, that this group of patients was prescreened for *GJB2* mutations, which account for up to 50% of all prelingual, non-syndromic HL cases in Caucasian populations [7][22]. Our diagnostic yield coincides well with results published in 2016 by Sloan-Heggen *et al*.[33], where they reported the analysis of the largest nsHL patient cohort to date with 1119 probands of mixed ethnicity, almost half of whom were of Caucasian origin. Their non-*GJB2* diagnostic yield reached 17.4%, which is comparable to the diagnostic yield in our nsHL study cohort. While several studies evaluated the diagnostic yield of non-syndromic HL [10], implementation of NGS technologies in syndromic HL has not been systematically studied. In the present group of 17 individuals with apparently syndromic HL, according to clinical examination and medical history, the disease-causing variant for HL was found in 8 individuals (47%). In a recent study, where children with syndromic HL had undergone genetic testing with a disease-targeted NGS panel for syndromic and non-syndromic HL genes, a diagnostic yield of 58.3% (28/48) was achieved, however the diagnosis of syndromic HL was not pinpointed solely on a clinical bases but determined after the results of genetic testing were known [34].

Second, clinical exome sequencing proved to be a useful tool in distinguishing between nsHL, sHL and HL as a separate feature of a complex phenotype.

Usually, when probands are found to have HL as the only presenting feature, a mutation in nsHL genes is suspected. However, in some syndromes special tests are required to detect secondary features or the penetrance of the secondary features is either incomplete or age dependent. These may lead to a false clinical categorization of patients with sHL into a group of apparently nsHL patients. In such cases the identification of the cause of HL would not be possible using disease-targeted panels for nsHL alone while clinical exome sequencing enables screening of non-syndromic and syndromic genes. One of the most common syndromic forms of HL, Usher syndrome, presents as a nsHL mimic early in life [33] as the onset of the secondary symptom (retinitis pigmentosa) does not appear until puberty. Two probands in the present cohort were found to have pathogenic *USH2A* variants; the older one (P852) presented with HL with retinal changes, while HL was the only clinical feature in the younger one (P584). Genetic testing enabled an early diagnosis of Usher syndrome, which is important in order to implement appropriate visual rehabilitation and to optimize learning and communication strategies. Waardenburg syndrome type 2A (OMIM193510), caused by pathogenic variants in the *MITF* gene, can also present as non-syndromic SNHL or as sHL with heterochromia iridum being the most common secondary feature. Two present probands had the same pathogenic variant in *MITF*. One presented as HL (P2091) and the other as HL with heterochromia (P045).

In patients with HL accompanied by clinical features in at least one other organ system, a syndromic diagnosis is suspected. As such, mutations of non-syndromic genes are often neglected during genetic testing. The clinical distinction between HL as a feature of a syndrome and HL as a separate feature of a complex phenotype can be difficult. The clinical exome sequencing approach can yield information concerning causes of nsHL, sHL, and a non-HL phenotype in the case of complex phenotypes. cES can aid in deciphering cases of syndromic HL (P584) and cases with complex phenotypes where HL is a separate feature (P144, P314, P555). As an example, proband P144, who suffered from mild high frequency HL and presented with clinical features of congenital myopathy, was found to have a novel, missense variant in a hotspot domain of the *RYR1* gene, explaining his primary diagnosis, but not the cause of HL. Proband P314, who was born after a twin pregnancy and presented with moderately severe HL, a complex clinical picture of major abnormalities (microphtalmia, solitary kidney, omphalocoele) and normal development, indicating a suspicion of a syndromic diagnosis. She was found to have a compound heterozygous variant in *GJB2*, explaining her HL, while the cause of the congenital anomalies remained unknown. Proband P555, with a family history of suspected Usher syndrome, was found to have the most common pathogenic homozygous variant in *GJB2*, while the cause of familial retinitis pigmentosa remained unknown as the father declined further testing. In families with complex pedigrees with several members presenting with HL, the application of trio analysis enabled us to detect that members from a single family had different genetic causes for HL (P453).

Lastly, 14 different non-*GJB2* HL disease causing variants in 10 genes (*CHD7*, *HDAC8*, *MITF*, *NEFL*, *OTOF*, *SF3B4*, *SLC26A4*, *TECTA*, *TMPRSS3*, and *USH2A*) were found among 13 patients confirming the extreme genetic heterogeneity of hereditary HL. Half (50%; 7/14) of the HL disease causing variants were novel and unique to a family, with only two variants in the *USH2A* and *MITF* genes, detected in two probands. Variant c.11864G>A in *USH2A* is one of the two most common pathogenic variants found in patients with Usher syndrome type 2A in Europe [28]. The distribution of the variant was shown to differ greatly among EU countries, with the lowest proportion found in France (4.5%) and the highest in Slovenia (82.5%) [28]. This coincides well with our findings of both patients with Usher syndrome having the homozygous c.11864G>A variant. Contrary to the small range of *USH2A* pathogenic variants, *MITF* pathogenic variants causing Waardenburg syndrome type 2 are diverse and usually private, with only a small number of exceptions [18], [35]. One of them is a pathogenic variant c.943C>T (p.Arg315*), which was detected in two unrelated families in our study, and has already been reported in three families originating from Northern Europe [16], India [17], and Togo [18].

The frequency of variants in some causative genes was high, with nearly half (6 of 13, 46%) of the non-*GJB2* causes attributable to four genes (*SLC26A4*, *USH2A*, *TECTA*, and *OTOF*). In the study by Sloan-Heggen et al. (2016) [33] these four genes were among the top 11 most common, non-*GJB2* identified genetic causes for hereditary HL (*STRC*, *SLC26A4*, *TECTA*, *MYO15A*, *MYO7A*, *USH2A*, *CDH23*, *ADCRV1*, *TMC1*, *PCDH15* and *OTOF)* that made up 67% of all successful diagnoses.

In summary, the clinical exome sequencing approach allowed us to comprehensively address the genetic heterogeneity of HL, to detect clinically unrecognized HL syndromes, and to distinguish between non-syndromic HL, syndromic HL, and HL as a separate feature of a complex phenotype.

## Supporting information

S1 Fig Pedigrees and sequence chromograms. (PNG)

S1 Table Clinical features of patients from syndromic hearing loss group. Fam, familial. HL, hearing loss. SNHL, sensorineural hearing loss. Spor, sporadic.
(PDF)

S2 Table 660 genes, associated with “hearing loss” HPO phenotypes. (PDF)
