# Supplementary figures and images for: Diagnostic outcomes of exome sequencing in patients with syndromic or non-syndromic hearing loss
Source: PLoS One. 2018 Jan 2;13(1):e0188578. doi: 10.1371/journal.pone.0188578 (PMC5749682; doi:10.1371/journal.pone.0188578)

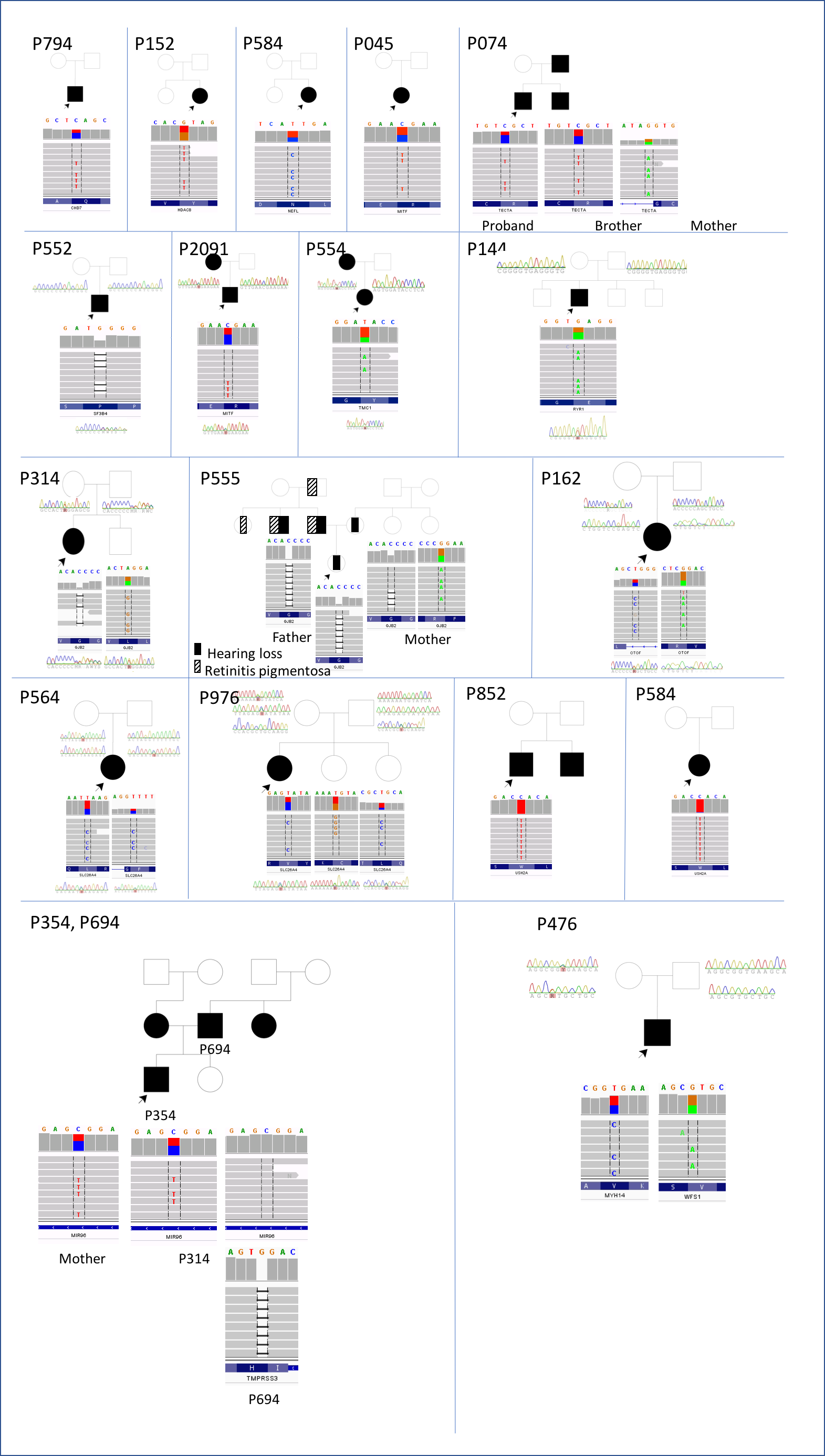

Supplement: S1 Fig — (PNG) [file pone.0188578.s001.png]
