# Supplementary material for: Diagnostic outcomes of exome sequencing in patients with syndromic or non-syndromic hearing loss
Source: PLoS One. 2018 Jan 2;13(1):e0188578. doi: 10.1371/journal.pone.0188578 (PMC5749682; doi:10.1371/journal.pone.0188578)
Supplement: S1 Table — Fam, familial. HL, hearing loss. SNHL, sensorineural hearing loss. Spor, sporadic. (PDF) [file pone.0188578.s002.pdf]

| Proband ID | Family history | Hearing loss | Other features |
|---|---|---|---|
| *P794* | *Spor* | *Progressive bilateral, mixed HL; SNHL component profound* | *External, middle and inner ear anomalies, myopia, bilateral retinal coloboma, hypothyroidism, coeliac disease* |
| *P152* | *Spor* | *Moderate bilateral SNHL* | *Global developmental delay, seizures, microcephaly, dysplastic facial features, fingers and toes syndactyly* |
| *P045* | *Fam* | *Severe bilateral SNHL* | *Unilateral heterochromia, dorsiflexion of 2nd toes* |
| *P584* | *Spor* | *Mild to moderate bilateral SNHL* | *Delayed motoric milestones, difficulties in walking, progressive distal weakness of the lower and upper limbs, cerebellar dysfunction, peripheral motor and sensory neuropathy* |
| *P144* | *Spor* | *Mild bilateral SNHL* | *Congenital arthrogryposis, delayed motoric milestones, severe scoliosis* |
| *P552* | *Spor* | *Moderately severe bilateral conductive HL* | *Malar hypoplasia, micrognathia, thumb hypoplasia, radioulnar synostosis* |
| *P555* | *Fam* | *Moderately severe bilateral SNHL* | *Both parents are hearing impaired, father also has RP along with some of his other close relatives* |
| *P314* | *Spor* | *Moderately severe bilateral SNHL* | *Microphthalmia, solitary kidney, omphalocele, dysplastic facial features; normal growth and development* |
| *P852* | *Fam* | *Moderate bilateral SNHL* | *Retinitis pigmentosa* |
| *P506* | *Fam* | *Severe bilateral SNHL* | *Severe developmental delay, cerebellar hypoplasia (vermis and hemispheres)* |
| *P654* | *Spor* | *Severe bilateral SNHL* | *Intestinal atresia type IIIa, unilateral hydronephrosis, developmental delay* |
| *P554* | *Spor* | *Moderate bilateral SNHL* | *Intellectual disability, autism, seizures, scoliosis, bronchogenic cyst of the neck* |
| *P052* | *Spor* | *Moderately severe bilateral conductive and SNHL* | *Aural atresia* |
| *P652* | *Spor* | *Moderate unilateral conductive and SNHL* | *Developmental delay, dysplastic facial features, choroidal coloboma, amblyopia, Dandy Walker malformation* |
| *P352* | *Fam* | *Profound bilateral SNHL* | *Hypopigmentation of hair* |
| *P952* | *Spor* | *Moderate unilateral conductive HL* | *Developmental delay, on the right side microtia (third degree), anophthalmia, and facial nerve paralysis, agenesis of corpus callosum* |
| *P752* | *Spor* | *Severe bilateral SNHL* | *Developmental delay, tracheoesophageal fistula, synostosis of the 5th and 6$^{th}$ rib on the right side, unilateral cryptorchidism* |
