# Supplementary material for: Diagnostic outcomes of exome sequencing in patients with syndromic or non-syndromic hearing loss
Source: PLoS One. 2018 Jan 2;13(1):e0188578. doi: 10.1371/journal.pone.0188578 (PMC5749682; doi:10.1371/journal.pone.0188578)
Supplement: S2 Table — (PDF) [file pone.0188578.s003.pdf]

AAAS, ABCA12, ABCA4, ABCB6, ABCC9, ABCD1, ABHD12, ABHD5, ACO2, ACOX1, ACTB, ACTC1, ACTG1, ACTN2, ACVR1, ACY1, ADAMTSL2, ADK, AIFM1, AIPL1, AK2, AKT1, ALG11, ALMS1, ALOX12B, ALOXE3, ALX3, AMER1, ANKH, ANKRD11, ANTXR1, AP1S2, ARHGEF6, ARID1A, ARID1B, ARL6, ARSB, ARSE, ARVCF, ASCL1, ASPA, ASPM, ASXL1, ATP1A2, ATP1A3, ATP6V0A4, ATP6V1B1, ATRX, B3GALTL, BAG3, BBS1, BBS10, BBS12, BBS2, BBS4, BBS5, BBS7, BBS9, BCAP31, BCOR, BCS1L, BDNF, BEAN1, BEST1, BMP15, BMP4, BRAF, BRCA2, BRIP1, BSND, BTD, BTK, BTRC, CA4, CACNA1A, CACNA1D, CASK, CCBE1, CCDC50, CD151, CDC6, CDH23, CDHR1, CDK5RAP2, CDT1, CEACAM16, CEP290, CEP57, CERKL, CHD7, CHN1, CHRNG, CHST14, CHST3, CHSY1, CIB2, CISD2, CLCN7, CLCNKA, CLCNKB, CLDN14, CLRN1, CNGA1, CNGB1, COA5, COCH, COL10A1, COL11A1, COL11A2, COL1A1, COL1A2, COL2A1, COL4A3, COL4A4, COL4A5, COL4A6, COL7A1, COL9A1, COL9A2, COL9A3, COLEC11, COMT, COQ2, COQ6, COX10, COX14, COX15, COX6B1, CRB1, CREBBP, CRX, CRYAB, CSRP3, CTC1, CTSA, DCAF17, DDX11, DES, DFNA5, DFNB31, DFNB59, DHCR7, DHDDS, DHODH, DIABLO, DIAPH1, DIAPH3, DKC1, DLX5, DMD, DMP1, DNAAF3, DNAI1, DNASE1L3, DNMT1, DOLK, DSG2, DSPP, DUX4, EBP, ECE1, EDN1, EDN3, EDNRB, EFNB1, EFTUD2, EHMT1, ELAC2, ERCC1, ERCC2, ERCC3, ERCC4, ERCC5, ERCC6, ERCC8, ESPN, ESRRB, EYA1, EYA4, EYS, FAM161A, FAM20C, FANCA, FANCB, FANCC, FANCD2, FANCE, FANCF, FANCG, FANCI, FANCL, FANCM, FARS2, FASTKD2, FBN1, FBXW4, FERMT1, FGF10, FGF3, FGF8, FGF9, FGFR1, FGFR2, FGFR3, FHL2, FIG4, FKBP14, FKTN, FLNA, FLNB, FMR1, FOXC1, FOXI1, FOXRED1, FRAS1, FREM2, FRG1, FSCN2, FSHR, FTO, FUCA1, G6PC3, GAA, GALC, GALE, GALNS, GATA1, GATA2, GATA3, GATAD1, GBA, GDF3, GDF5, GDF6, GDNF, GFER, GIPC3, GJA1, GJB1, GJB2, GJB3, GJB4, GJB6, GJC2, GLA, GLB1, GLYCTK, GNAS, GNS, GP1BB, GPC3, GPR98, GPSM2, GRHL2, GRIP1, GRXCR1, GSC, GUCA1B, GUCY2D, GUSB, HARS, HARS2, HBB, HCCS, HDAC4, HDAC8, HESX1, HEXA, HEXB, HGF, HGSNAT, HNF1B, HOXA1, HOXA11, HOXA2, HS6ST1, HSD17B10, HSD17B4, IDH3B, IDS, IDUA, IGBP1, IGF1, ILDR1, IMPAD1, IMPDH1, IMPG2, IQCB1, IRX5, ITM2B, KAL1, KARS, KAT6B, KCNE1, KCNJ10, KCNJ13, KCNQ1, KCNQ4, KDM6A, KIF7, KISS1R, KIT, KLHL7, KMT2D, KRAS, KYNU, L2HGDH, LAMA4, LARS2, LCA5, LDB3, LEMD3, LHFPL5, LHX3, LMNA, LMNB1, LMX1B, LOXHD1, LRAT, LRP2, LRP4, LRP5, LRTOMT, LZTFL1, MAK, MAN2B1, MANBA, MARS2, MARVELD2, MASP1, MBTPS2, MED12, MERTK, MFN2, MGAT2, MGP, MIR96, MITF, MKKS, MKS1, MOGS, MPZ, MSRB3, MYBPC3, MYCN, MYD88, MYH14, MYH3, MYH6, MYH7, MYH9, MYO15A, MYO1A, MYO3A, MYO6, MYO7A, MYPN, NAA10, NAGA, NAGLU, NDP, NDRG1, NDUFA1, NDUFA10, NDUFA11, NDUFA12, NDUFA2, NDUFA9, NDUFAF1, NDUFAF2, NDUFAF3, NDUFAF4, NDUFAF5, NDUFAF6, NDUFB3, NDUFB9, NDUFS1, NDUFS2, NDUFS3, NDUFS4, NDUFS6, NDUFS7, NDUFS8, NDUFV1, NDUFV2, NEU1, NEXN, NF2, NFIX, NHP2, NIPAL4, NIPBL, NLRP12, NLRP3, NMNAT1, NOG, NOP10, NOTCH2, NOTCH3, NPHP1, NR2E3, NR5A1, NRAS, NRL, NRTN, NSD1, NSDHL, NSMF, NUBPL, OFD1, OPA1, ORC1, ORC4, ORC6, OTOA, OTOF, PALB2, PAX1, PAX2, PAX3, PCDH15, PCNT, PDE4D, PDE6A, PDE6B, PDE6G, PDZD7, PEPD, PEX1, PEX10, PEX11B, PEX12, PEX13, PEX14, PEX16, PEX19, PEX2, PEX26, PEX3, PEX5, PEX6, PEX7, PHEX, PHF6, PHOX2B, PHYH, PIEZO2, PIGA, PIGL, PIGV, PIK3CA, PIK3R1, PITX2, PLA2G6, PLN, PLOD3, PLP1, PMP22, PNPLA1, PNPLA2, POLD1, POLG, POLR1C, POLR1D, POR, PORCN, POU3F4, POU4F3, PQBP1, PRCD, PRDM5, PRKAR1A, PRKDC, PROK2, PROKR2, PROM1, PRPF3, PRPF31, PRPF6, PRPF8, PRPH2, PRPS1, PRRT2, PRRX1, PSEN1, PSEN2, PSMC3IP, PTCH1, PTCH2, PTEN, PTH1R, PTPN11, PTPN22, PTPRQ, RAB23, RAD21, RAD51C, RAF1, RAI1, RBM20, RBM8A, RBP3, RD3, RDH12, RDX, RECQL4, RET, RGR, RHO, RLBP1, RNASET2, ROBO3, ROM1, ROR2, RP1, RP2, RP9, RPE65, RPGR, RPGRIP1, RPS6KA3, RRM2B, RUNX2, SAG, SALL1, SALL4, SBF2, SCN1A, SCN5A, SDCCAG8, SDHA, SDHB, SDHC, SDHD, SEMA3A, SEMA3E, SEMA4A, SERPINB6, SETBP1, SETD2, SF3B4, SGCD, SGSH, SH3TC2, SHANK3, SHFM1, SHOC2, SIX1, SIX5, SKI, SLC17A8, SLC19A2, SLC25A4, SLC26A2, SLC26A4, SLC26A5, SLC29A3, SLC33A1, SLC4A11, SLC52A3, SLX4, SMAD4, SMARCA4, SMARCB1, SMARCE1, SMC1A, SMC3, SMPX, SNAI2, SNAP29, SNRNP200, SNX10, SOS1, SOST, SOX10, SOX2, SOX9, SPATA7, SPTLC1, SQSTM1, SRCAP, ST3GAL5, STRC, SUCLA2, SUCLG1, SUFU, SUMF1, SURF1, SYT2, TACO1, TACR3, TAZ, TBC1D24, TBX1, TBX22, TCAP, TCIRG1, TCOF1, TECTA, TERC, TERT, TFAP2A, TFAP2B, TGFB1, TGM1, THRB, TIMM8A, TINF2, TK2, TMC1, TMIE, TMPO, TMPRSS3, TNC, TNFRSF11A, TNFRSF11B, TNFSF11, TNNC1, TNNI3, TNNT2, TOPORS, TP63, TPM1, TPRN, TRIM32, TRIOBP, TRPV3, TRPV4, TTC19, TTC8, TTN, TTR, TULP1, TWIST1, TXNRD2, TYMP, TYR, UBR1, UFD1L, UGT1A1, USB1, USH1C, USH1G, USH2A, VCL, VHL, VIPAS39, VPS13B, VPS33B, WDPCP, WDR11, WFS1, WNT10B, WNT5A, WRAP53, XPA, XPNPEP3, YAP1, ZNF469, ZNF513.
